# Dendritic Cell and T Cell Crosstalk in Liver Fibrogenesis and Hepatocarcinogenesis: Implications for Prevention and Therapy of Liver Cancer

**DOI:** 10.3390/ijms21197378

**Published:** 2020-10-06

**Authors:** Isabella Lurje, Linda Hammerich, Frank Tacke

**Affiliations:** Department of Hepatology and Gastroenterology, Campus Virchow-Klinikum, Charité University Medicine Berlin, 13353 Berlin, Germany; Isabella.Lurje@charite.de (I.L.); Linda.Hammerich@charite.de (L.H.)

**Keywords:** HCC, fibrosis, cirrhosis, dendritic cells, T cells, tumor tolerance, antigen-presenting cells, immunotherapy, checkpoint inhibitors, dendritic cell vaccine

## Abstract

Liver fibrosis is a chronic, highly prevalent disease that may progress to cirrhosis and substantially increases the risk for development of hepatocellular carcinoma (HCC). Fibrotic livers are characterized by an inflammatory microenvironment that is composed of various immunologically active cells, including liver-resident populations (e.g., Kupffer cells, hepatic stellate cells and sinusoidal endothelium) and infiltrating leukocytes (e.g., monocytes, monocyte-derived macrophages, neutrophils and lymphocytes). While inflammatory injury drives both fibrogenesis and carcinogenesis, the tolerogenic microenvironment of the liver conveys immunosuppressive effects that encourage tumor growth. An insufficient crosstalk between dendritic cells (DCs), the professional antigen presenting cells, and T cells, the efficient anti-tumor effector cells, is one of the main mechanisms of HCC tumor tolerance. The meticulous analysis of patient samples and mouse models of fibrosis-HCC provided in-depth insights into molecular mechanisms of immune interactions in liver cancer. The therapeutic modulation of this multifaceted immunological response, e.g., by inhibiting immune checkpoint molecules, in situ vaccination, oncolytic viruses or combinations thereof, is a rapidly evolving field that holds the potential to improve the outcome of patients with HCC. This review aims to highlight the current understanding of DC–T cell interactions in fibrogenesis and hepatocarcinogenesis and to illustrate the potentials and pitfalls of therapeutic clinical translation.

## 1. Introduction

The liver is not only important for metabolism, detoxification and protein synthesis, but also contains many immune cells that control homeostasis and defense against pathogens. The immunological landscape of the liver is shaped by continuous exposure to non-self antigens from the portal venous blood that would ordinarily provoke an immediate immune response. An intense immunological interaction is facilitated by a slow blood flow in the liver sinusoids, their lining by specialized liver sinusoidal cells (LSECs) and a fenestrated endothelium that enables the contact to the underlying space of Disse, and thus, hepatocytes [1]. A plethora of resident and non-resident antigen-presenting cells (APCs) and adaptive immune cells orchestrate the unique hepatic milieu of tolerance to antigens from nutrients or resident microbiota while maintaining the possibility of swift and vehement responses against infections and tumors [2]. In this regard, the interaction of dendritic cells (DCs) and T cells constitutes a central axis that, together with macrophages, monocytes and innate lymphoid cells, regulates the tolerogenic or immunogenic direction of the immune answer [3]. In the setting of hepatic diseases, liver immunity is not only transformed, but it also exerts an immense influence on the progression of disease [4,5] and its dysfunction is considered as a perpetuator of liver fibrosis and tumorigenesis [1].

Hepatic fibrosis and cirrhosis constitute a major source of morbidity and mortality worldwide, with viral hepatitis, alcohol-related liver disease (ALD) and nonalcoholic steatohepatitis (NASH) constituting the most common etiologies [6]. Fibrosis and, later, cirrhosis evolve in the course of chronic liver injury, when the physiological parenchymal structure is progressively supplanted by fibrotic septa that subdivide the liver into regenerative nodules of hepatocytes. These morphological changes originate from hepatic stellate cell (HSC) activation and their transdifferentiation into myofibroblasts, causing an overproduction of extracellular matrix (ECM) and fibrogenesis, increased vascular resistance and amplification and dysregulation of inflammatory responses [7].

Hepatocellular carcinoma (HCC) is the most common primary liver tumor and typically develops in the context of liver fibrosis or cirrhosis. The incidence of HCC in cirrhotic patients is between 2 and 7% per year, depending on the etiology of the chronic liver disease [8]. Generally, the incidence of HCC is rising in many regions, and the majority of HCC diagnoses are made in stages of disease not amenable to curative treatments, which comprise of orthotopic liver transplantation, liver resection or tumor ablation [9,10]. At the same time, the options of interventional and medical therapy are limited by the underlying liver disease and the chemoresistance of HCC [11]. Multikinase inhibitors such as Sorafenib were celebrated as the first description of an efficient systemic therapy in advanced HCC but could only prolong overall survival (OS) by less than three months [12,13,14]. First reports of the immune checkpoint inhibitor Nivolumab in HCC therapy showed response rates of 15–20% and stable disease in 45% of patients [15,16]. The response rate is closely linked to the immune status of the tumor, especially to the exhaustion of the cytotoxic T cell response [17]. Studies aiming to overcome this blockade and to investigate the role of tumor immunology in the development of new therapeutic strategies are eagerly awaited. At present, the combination of the immune checkpoint inhibitor atezolizumab and the antiangiogenic antibody bevacizumab are considered the most effective systemic therapy in advanced HCC, emphasizing the prominent role of immune-directed therapies in the treatment of liver cancer [18].

The last years have witnessed a rapid development of therapeutic strategies specifically targeting communication of APCs, T cells and tumor cells, justifying an intensified interest in the DC T cell axis. A such, the interaction of DCs, as the most potent APCs, can mediate a tolerogenic or immunogenic direction of the T cell response, while CD8+ T cells, once activated, constitute the most effective antitumor defense. Here, we will focus on the DC–T cell axis and describe other cellular compartments if they pertain to the general understanding of liver physiology or disease or if they influence the antigen-presenting axis. This review aims to give a comprehensive overview of the role of DC and T cell interaction in liver cirrhosis and hepatocarcinogenesis in the context of new therapeutic opportunities to overcome the immune tolerance of HCC and to improve patient outcomes.

## 2. Crosstalk of DCs and T Cells

### 2.1. DCs in the Liver

Since the first morphological report of a cutaneous DC type of unknown function by Langerhans in 1868 and the description of DCs by Steinman and Cohn in peripheral lymphoid organs in 1973, it has been recognized that DCs form an essential link between innate and adaptive immunity [19,20]. As such, DCs are capable of antigen presentation, govern the subsequent differentiation of T cells and regulate T cell responses [21]. In contrast to macrophages, DCs have migratory properties and usually present antigen to T cells in the tissue-draining lymph node(s) [22]. The liver is different from other tissues in this regard, as many DC–T cell interactions seem to directly occur in the liver [23].

Human DCs commonly express the major histocompatibility complex (MHC) molecule, human leukocyte antigen (HLA)-DR, cluster of differentiation (CD) 209 and integrin CD11c, which can also be found on subsets of macrophages, activated T cells, B cells and natural killer (NK) cells [3]. There is a great heterogeneity of DC populations, depending on the developmental lineage, differentiation stage and the influence of surrounding tissue [24]. The main subsets of DC are generally distinguished by the combination of the four markers BDCA-1 (CD1c), BDCA-2 (CD303), BDCA-3 (CD141) and BDCA-4 (CD304). Conventional DCs (cDCs), formerly sometimes called myeloid DCs, can be divided into CD141+/CD14- type 1 cDCs (cDC1) and CD1c+/CD14− type 2 cDCs (cDC2). The latter are the most frequent DC type in the human liver but are rarely encountered in peripheral blood or spleen [25]. Plasmacytoid DCs (pDCs) express CD303 and CD304, orchestrate antiviral responses and characteristically secrete type 1 interferon (IFN), stimulating NK cells, B cells, T cells and myeloid DCs [26]. In chronically inflamed environments, monocytes can differentiate into inflammatory DCs (infDCs), a subset that can induce T helper cell (Th) 17 cell differentiation from naive CD4+ T cells. The developmental pathway of infDCs shares characteristics of both DC and macrophage development and features of in vitro-generated monocyte-derived DCs (moDCs) [27]. Novel technologies like single-cell RNA sequencing and single-cell protein analyses have further refined our perception of DC heterogeneity by demonstrating that the cDC2 subtype can be further subdivided into distinct groups, including a circulating inflammatory subset, and allowing for a clear differentiation between monocytes and cDC2 [28]. The frequency of DCs is similar in murine and human livers, but their subset marker expression varies considerably between the two species (Table 1) [3].

In 2013, Ibrahim et al. suggested a compelling classification of liver DCs based on DC lipid content and lipid metabolism. A DC population with high lipid concentrations (high-DC) that activated T cells, NK cells and NKT cells, exhibited a high MHC class II (MHCII) and toll-like receptor (TLR) expression accounted for approximately 75% of liver DCs, whereas a second DC population with low levels of lipids (low-DC) induced regulatory T cells, anergy to cancer and oral tolerance [29].

### 2.2. T Cells in the Liver

The hepatic composition of lymphocytes differs from blood and other lymphoid organs. The liver contains a large number of “unconventional” lymphocytes, including innate and innate-like lymphocytes, mucosa-associated invariant T cells, γδ T cells, NK and NKT cells, alongside classical components of the adaptive immunity such as T- and B-cells [3]. With respect to classical T cells, peculiarities in the human liver include a reversed CD4/CD8 ratio (1:3.5) compared with that in peripheral blood (2:1) and an increased double positive CD3+ CD4+ CD8+ lymphocyte population [34]. The hepatic T cell landscape develops through the attraction of certain lymphocyte types from the liver sinusoids, where LSECs constitutively express a high density of the adhesion molecules intercellular adhesion molecule (ICAM) 1, ICAM2 and vascular adhesion protein 1 (VAP1) that engage with activated T cells and CD8+ T cells and NK cells [1,35]. Additionally, the hepatic immune landscape is enriched for NKT cells and special subsets of T cells [34].

### 2.3. DC and T Cell Interaction in the Liver

Circulating DCs, monocytes and other DC precursors migrate through the liver, exerting phagocytic activity and then move from the sinusoidal area through the hepatic lymph tracts to the locoregional lymph nodes [36]. In the sinusoids, DCs can bind selectively to Kupffer cells via lectin-like receptors, an interaction that seems to be essential for DC recruitment and adherence to the sinusoidal walls and the subsequent MHC I-restricted cross-presentation of Kupffer cell-derived antigens by DCs [37]. The interaction between Kupffer cells and DCs is mediated by the secretion of CCL3 by Kupffer cells in response to inflammatory signals, which interacts with CCR1 on immature DCs [1].

On the way to the lymph node DC mature, reflected by their expression of CCR7 and the loss of CCR1 and CCR5 [1]. Through MHC II-restricted antigen presentation, DCs (similarly to Kupffer cells and LSECs) can interact with CD4+ T cells to present extracellular antigens. Intracellular antigens are processed either through the cytosolic or the vacuolar pathway. The former involves proteasomal degradation and the subsequent transporter associated with antigen processing (TAP)-dependent loading of peptides on MHC class I molecules in the endoplasmic reticulum or endosomes/phagosomes [38]. After activation by a CD4+ T cell, cDC1 and pDCs can then mediate licensing, transferring MHCI-mediated activation signals to a CD8+ T cell and inducing effector and memory CD8+ T cell formation [32,39].

The hepatic innate immune response is influenced by a chronic low-level exposure to endotoxins (lipopolysaccharides, LPSs) from the portal blood. This leads to the desensibilization against LPS, the downregulation of responses to infective triggers and the secretion of interleukin (IL)-10 by Kupffer cells [40]. A similar mechanism was unveiled regarding cDC2, the most prevalent DC subset in the liver. When executing their regular function of Th cell priming, their stimulation through TLR 4 resulted in the secretion of IL-10, but comparatively low levels of proinflammatory cytokines [25]. Antigen presentation with concomitant tolerogenic signals is crucial to the induction of T cell hyporesponsiveness in the homeostatic setting [25,30]. Thus, the triggering of a peptide-specific anergy in various CD4+ and CD8+ T cell populations is the ordinary response to hepatic antigen presentation [41]. An increased extra- and intracellular cytotoxic T-lymphocyte antigen (CTLA)-4 expression has been observed in these anergic T cells [41]. In turn, anergic CD4+ and CD8+ T cells suppress the proliferation of syngeneic T cells through cell-to-cell contacts [41]. CTLA-4 furthermore binds and removes costimulatory molecules CD80 and CD86 from APCs, including DCs, thus hampering their antigen-presenting function and CD4+ T cell activation [42]. A resulting upregulation of indoleamine 2,3-dioxygenase (IDO) induces a regulatory, tolerogenic phenotype in pDCs and moDCs, which in turn transforms naïve T cells into immune-suppressive regulatory T cells (Tregs) [43,44], substantiating the observation that isolated hepatic DCs are capable of inducing Tregs from naive CD4+ precursors [25]. However, it should be noted that Kupffer cells, the resident phagocyte population of the liver, are also capable of inducing Tregs in healthy liver, as exemplarily shown for tolerance induction against particulate antigens [45].

## 3. Fibrosis and Cirrhosis

Liver fibrosis constitutes the major complication of chronic liver disease that can ultimately progress to liver cirrhosis—a fibrotic remodeling with a distortion of the vasculature and the development of regenerative nodules. The major complications of cirrhosis include an impaired hepatic function and severe systemic effects such as portal hypertension, hepatic encephalopathy and disturbances of blood coagulation and an increased risk to develop HCC [8,46]. Characteristically, fibrosis evolves when damaged tissue is replaced by ECM-rich scar tissue as a part of the hepatic wound healing response [8]. Central to this mechanism is the activation of HSC into proliferative, contractile and fibrogenic myofibroblasts through signals like endothelial fibronectin release, mediators from apoptotic or necrotic hepatocytes, platelet-derived mediators like the platelet-derived growth factor (PDGF), transforming growth factor (TGF)-β, epidermal growth factor (EGF) and profibrogenic signals from liver macrophages such as TGF-β and reactive oxygen species (ROS) [47]. The concomitant activation of Kupffer cells, HSCs and endothelial cells results in an upregulation of adhesion molecules such as E-Selectin, ICAM-1 and vascular cellular adhesion molecule (VCAM)-1 and facilitates the adhesion of APCs and T cell trapping [3].

Hepatic macrophages are well studied in chronical liver disease, and they have an ambiguous role, acting both as activators of HSC but also governing tissue restoration and resolution of fibrosis. In chronical liver injury, the liver macrophage pool is enriched through an enhanced monocyte recruitment from blood and a local differentiation from CD14^high^CD16− classical monocytes [48].

Two main systemic immunological alterations are typical in the setting of cirrhosis. On the one hand, cirrhosis-associated immune dysfunction results in an immunodeficiency that becomes evident in increased susceptibility to bacterial infection and impaired immunosurveillance [49]. On the other hand, the persistent inadequate stimulation of cells of the immune system leads to systemic inflammation [49].

### 3.1. DCs in Liver Fibrosis

Concerning the role of DCs in the setting of chronic liver inflammation and fibrosis, several, in part contradictory, hypotheses have been brought forward, ranging from a protective and restorative function to an aggravation of inflammation [50,51]. Part of this controversy is certainly related to difficulties in defining DCs in the liver, since monocytes, macrophages and Kupffer cells can express “DC markers” in the liver (e.g., CD11c and MHCII in mice), making it challenging to dissect DC from traditional phagocyte functions [52]. This is even more difficult in conditions of diseased liver, in which the myeloid compartment is largely augmented by infiltrated monocyte-derived cells, including monocytic subsets with a clear differentiation towards DCs. While it is conceivable that DCs execute various roles depending on the etiology and the chronic or acute nature of the liver disease, the presence of concomitant activation signals and the overall immunological milieu, some of the available studies may need to be re-examined to clarify their exact implications [51].

In mouse models of carbon tetrachloride (CCl_4_)-induced acute liver injury and methionine/choline-deficient (MCD)-diet induced steatohepatitis, disease progression was accompanied by a substantial expansion of CD11c+/MHCII^high^/CD11b+ fractalkine receptor (CX3CR1) expressing cells that were identified as moDCs. Mice with a defective maturation of monocytes into moDCs and mice treated with a CX3CR1 antagonist showed an attenuation of hepatic inflammatory injury [53]. Similarly, tumor necrosis factor (TNF)α-producing CX3CR1+ moDC depletion ameliorated hepatocellular inflammation in the NASH mouse model, thus suggesting a proinflammatory role of moDCs in steatohepatitis progression [54].

In 2009, a study by Connoly et al. reported an enhanced DC immunogenicity in hepatic fibrosis that was deduced from the ability of CD11c+ cells to activate T cells and NK cells and to produce TNFα [55]. The authors noted a significant expansion of CD11c+ DCs, Gr1+CD11b+ myeloid cells and CD8+ T lymphocytes in this murine fibrosis model, while the percentage of liver CD4+ T lymphocytes was significantly lower than in nonfibrotic controls. More specifically, fibrotic livers showed a significant increase in CD11b+CD8- DCs with a more mature phenotype than in normal livers [55]. However, as mentioned above, CD11c+ macrophages and monocytes are also present in chronic liver inflammation, which would be included in the analysis when simply gating on CD11c+ cells, a fact that may have contributed to the overall proinflammatory role attributed to liver DCs in this study. Monocytes have been previously described as perpetuators of inflammation and triggers of fibrotic remodeling [56]. In a CD11c-diphteria toxin receptor (DTR) mouse model, depletion of CD11c+ cells resulted in a lower cytokine and chemokine production, further corroborating a proinflammatory role for DCs. [51].

Conversely, Pradere and colleagues reported that liver macrophages, but not DCs, promoted fibrosis by enabling the survival of activated HSCs/myofibroblasts via the NF-κB pathway in bile duct ligation (BDL)- and CCl_4_-induced murine liver fibrosis models. In coculture experiments, the authors observed that CD11c+ DCs induced a moderate upregulation of NF-κB in HSCs via TNF and IL-1 production at considerably lower levels than hepatic macrophages and did not mediate HSC activation. Ablation of cDCs or pDCs in vivo did not affect liver fibrosis, leading to the conclusion that neither cDCs nor pDCs significantly contribute to liver fibrogenesis in vivo [57].

In 2014, Blois et al. showed that the antifibrotic role of DCs is closely linked to antiangiogenetic effects. The working group reported on a thioacetamide liver fibrogenesis model in which DC depletion in CD11c+ DTR mice accelerated the development of fibrosis and resulted in the overproduction of angiogenic growth factors. Thus, during chronic thioacetamide exposure, an enhanced expression of the antiangiogenic soluble vascular endothelial growth factor (VEGF) receptor 1 (sFlt-1) was noted on hepatic DCs, thus decreasing the bioavailability of VEGF. In CD11c+ DC depleted-mice, recombinant Flt-1 conveyed a protective response against fibrosis progression [58]. Interestingly, these DC functions apparently counteract proangiogenic signals that have been observed from monocyte-derived macrophages in mouse models of liver fibrosis and fibrosis-driven HCC [59,60].

When analyzing the regression phase of a CCl_4_ murine fibrosis model, Jiao et al. noted a delayed regression of fibrosis and a persisting population of activated HSC when CD11c+ DCs were depleted. In line with that, increasing the numbers of DCs through in vivo expansion or adoptive transfer resulted in matrix metalloproteinase (MMP)9-dependent resolution of fibrosis [50]. This process was independent of NK cells, whose antifibrotic role by means of killing activated HSCs and secreting IFN-γ is well established [50,61].

### 3.2. T Cells in Liver Fibrosis

While the pathophysiological role of the innate immune system in fibrogenesis and cirrhosis is well described, especially for HSC and hepatic macrophages, less studies are available on the exact role of the adaptive immune system. However, there is an emerging body of evidence describing important contributions of adaptive immune cells to chronic liver diseases and fibrosis [62,63]. Several studies point towards a profibrotic role of the T cell response whilst the progression of cirrhosis in turn disrupts T cell function [49,64].

CD8+ effector T cell trafficking to the liver is independent of classical adhesion molecules and hepatic inflammation but is mediated by platelet adherence to sinusoidal hyaluronan via CD44. Then, CD8+ effector T cells actively crawl along the sinusoidal endothelial lining, extending protrusions through endothelial cell fenestrae to interact with the hepatocyte membrane [65].

In contrast to other non-lymphoid organs, priming of circulating naïve CD8+ T cells can also occur in the liver, with CD8+ T cells directly interacting with MHC I complexes on hepatocytes [66]. CD8+ T cells primed by hepatocytes, the main target of hepatotropic viruses, do not differentiate into effector cells and are unresponsive to checkpoint inhibition [67].

A polarization towards Th2 and Th17 responses has been suggested as a driving force of hepatic fibrosis. While Th2 responses convey an anti-inflammatory effect via IL-13 and TGF-β, they have also been identified as a stimulator of ECM remodeling and collagen deposition in chronic irritants [68]. Studies in schistosomiasis-infected cytokine-deficient mice demonstrated that a CD4+ Th2 cell response with a dominant profibrotic role of IL-13, and not a Th1 response, was conductive to the development of liver fibrosis [69], but also to the immunological control of the parasite [70,71]. The stimulation of macrophages to produce TGF-β1, which in turn triggers myofibroblast activation, seems to be a central mechanism in this regard [72].

Type 3 inflammation, characterized by a polarization towards a Th17 response and the production of IL-17A, IL-17F, IL-22 and IL-26 by Th17 cells, Th 22 cells, neutrophils and mast cells has been identified as a driver of chronical liver injury and of TGF-β-dependent liver fibrosis [73]. An increased proportion of Th17 cells and increased IL-17 plasma levels have been described in patients with various etiologies of chronic liver disease, such as alcoholic liver disease, NASH and viral infection [74,75,76]. While hepatic infiltration with IL17-secreting cells has been noted in most chronical cirrhosis etiologies, this effect was especially pronounced in alcoholic and immune hepatitis. IL-17 stimulation induces HSCs to recruit neutrophils through IL-8 and growth-related oncogene α (GRO-α) secretion in vitro, suggesting that Th17 polarization perpetuates inflammation and chronic liver injury [74]. Furthermore, IL-17 plays a critical role in the pathogenesis of cholestatic and hepatotoxic liver fibrosis in mice. The pathogenetic mechanisms of IL-17 signaling include both the stimulation of Kupffer cells to express inflammatory and profibrotic cytokines, and Stat3-dependent HSC activation [77]. At the same time, IL-22 exerts a profibrotic function through enhancement of TGF-β signaling in HSC [73]. Blocking type 3 inflammation, either by IL-17+ cell depletion or by inhibition of IL17/IL22 production reduced the degree of liver fibrosis and prevented fibrosis progression in mouse fibrosis models of different etiologies [73,75].

The ability of Tregs to terminate immune responses results in two distinct effects on liver fibrogenesis versus the resolution of fibrosis [78]. Tregs have been shown to secrete IL-10, thus preventing immunogenic Kupffer cells from inducing effective CD8+ cytotoxic T cells [79]. Furthermore, Tregs significantly inhibited IL-17 and IL-22 cytokine production, thus abrogating the profibrinogenic effects of Th17 cells and inhibiting HSC activation [75]. In contrast, the immunosuppressive effects of Tregs impaired the resolution of fibrosis by inhibiting Kupffer cell MMP production and prevented the resolution of fibrosis in a murine CCl_4_ fibrosis model. Treg depletion resulted in decreased TGF-β, MMP9 and tissue inhibitor of metalloproteinases (TIMP)1 levels, increased expression of MMP2 and MMP14 and a resolution of fibrosis [80]. It was further noted that Tregs shifted the Kupffer cell polarization towards an anti-inflammatory (“M2-like”) phenotype [81]. In turn, Kupffer cells, and LSECs and HSCs, are capable of priming naive CD4+ T cells towards a Treg-cell phenotype [3].

While the role of NK cells as antifibrotic cells that remove activated HSCs and produce IFN-γ has been clearly delineated, NKT cells have been shown to both produce profibrotic cytokines (IL-4 and IL-13) and to have an antifibrotic role in removing activated HSCs [61]. After induction of liver injury in mice, NKT cell accumulation is an early event that promotes sustained tissue inflammation [82]; this inflammatory activity, however, also helps to recognize and remove senescent hepatocytes as a tumor-preventive immune surveillance mechanism [83]. An accumulation and activation of intrahepatic CD4+ T cells, CD8+ T cells, regulatory T cells and NKT cells has been observed in NASH models of mice fed with a MCD-diet. CD8+ T cells showed an increased TNF secretion, while CD4+ T cells secreted more IL-17 than in healthy controls. CD8+ T cells and NKT cells cooperatively caused the progression of NASH. In this setting, NKT cells enhanced the fatty acid uptake by hepatocytes. CD8+ T cell and NKT cell-deficient mice did not develop NASH when fed with an MCD diet, while CD8+ T cell depletion in mice with manifest NASH led to a reversal of liver injury. In comparison to healthy controls, patients with chronic liver disease of various etiologies (ALD, NASH, Hepatitis C and HCC) had significantly increased numbers of CD8+ T cells, while an increased number of CD57+CD3+ NKT cells was noted only in NASH and viral hepatitis. Furthermore, CD8+ T cells and NKT cells as mediators of chronic hepatic injury were implicated in NASH-to-HCC progression [84].

Hepatic fibrosis due to viral hepatitis has the distinctive feature of an antigen response that is directed immediately against specific viral antigens, while the underlying mechanism of most other etiologies (e.g., NASH) is an unspecific inflammatory response [85]. In chronic viral hepatitis, liver injury is furthered both by virus-specific and nonspecific T cells and modulated by suppressor cells, whose function, in turn, is altered in the setting of chronic viral infection. The DC–T cell communication in antiviral responses is both a determinant of liver injury and of virus elimination, and has been described in detail elsewhere [5,86,87].

Although manifest cirrhosis is primarily characterized by activated innate immune mechanisms [88], liver cirrhosis also profoundly affects the function and frequency of T and B lymphocytes [49]. A diminished B cell population has been reported in viral and alcoholic etiologies of cirrhosis [49,89]. A significant loss of CD27(+) IgM(+) memory B cells, which mediate T cell-independent immunity, has been noted in both viral and non-viral cirrhosis with and without HCC, while their numbers were largely unchanged in early fibrosis [90,91]. Simultaneously, a significant increase in CD27+CD38hi plasmablasts was observed among cirrhotic patients. Furthermore, B cells from cirrhotic patients were hyporesponsive to strong activation stimuli (CD40/TLR9) as reflected in the impaired upregulation of CD70 and TNFβ and in a lower IgG production. The presence of cirrhosis was also associated with a lower B cell allostimulatory capacity, manifested in the reduced ability of alloreactive CD4+ T cells to produce TNFα and TNFβ after B cell contact [91]. The number and composition of CD4+ T cells was also affected in cirrhosis of various etiologies: lowered CD4+ T cells counts were associated with splenomegaly, thrombocytopenia and leukopenia [92]. The depletion of naive Th cells was found to be more pronounced than of the memory cell compartment. The underlying mechanisms were identified as a defect in thymopoiesis, splenic pooling and activation-induced cell-death induced by bacterial translocation [93].

## 4. Carcinogenesis and HCC

### 4.1. DCs in HCC

Dysfunctional antigen presentation is a central component of an impaired cytotoxic T cell response. As such, DCs as potent professional APCs constitute a central topic of research. Generally, the main mechanisms by which DCs contribute to tumor growth in various tumors can be summarized as: (i) dysfunction/lipid overload, (ii) induction of tolerance to tumor antigens and (iii) suppression of T cell function through mediator release or checkpoint ligand expression. Several of these mechanisms have been identified in human HCC samples and mice models (ii–iii), however, specific data on DC dysfunction by lipid overload in HCC is scarce (i).

(i) DC dysfunction has been described in tumor-bearing mice and patients with various tumors, who exhibit DCs with high amounts of triglycerides, which fail to stimulate allogeneic T cells and are unable to present tumor-specific antigens. Inhibition of acetyl-CoA carboxylase to normalize the DC lipid content resulted in a restoration of their antigen-presenting, immunostimulatory function [94]. In contrast to physiologically resident hepatic high-DC populations with an elevated endogenous lipid content that convey an immunostimulatory effect [29], dysfunctional DCs showed an upregulated scavenger receptor A-mediated uptake of extracellular lipids, a larger size of lipid bodies and an altered lipid composition with an accumulation of oxidatively truncated lipids [94,95]. Through the binding of chaperone heat shock protein 70, oxidatively truncated lipids prevent the translocation of peptide-MHCI to the cell surface, thus inhibiting cross-presentation while not affecting endogenous antigen presentation [95,96]. This effect could be reversed by the incubation of DCs with tumor explant supernatant, resulting in a downregulation of peptide-MHC [94].

(ii) Cancer cells can induce an immature differentiation state of DCs by downregulating antigens and adhesion molecules or by secreting immunosuppressive factors like IL-10 and VEGF [97,98]. Immature DCs facilitate tumor tolerance by inducing antigen-specific CD8+ Tregs, suppressing the function of other effector T cells [99]. A further pathological activation state, termed “semi-mature”, develops after contact with dying cancer cells, typically in the context of non-immunogenic anticancer treatment. Semi-mature DCs are able to sustain only 2 of 3 T cell activation signals, and their unstable engagement with T cells encourages T cell anergy or T cell exhaustion [98,100].

The effect of tumor immunogenicity, reflected in adhesion molecule expression, has been studied in hepatoma cell lines. As such, hepatoma cell lines with high adhesion molecule expression induced a tolerogenic pDC subset (CD11c^high^ MHC-II^high^ DEC-205+) with impaired cross-presenting capacities and low costimulatory molecule expression, resulting in an impaired activation of CD8+ cytotoxic T cells [97].

Conversely, mature DCs do not only induce antitumor reactive T cells but can also mediate HCC immune evasion by inducing tolerogenic responses to presented tumor antigens. The contact of mature DCs with tumor-derived factors can induce Treg1-like cells from naive CD4+ T cells through high IL-10 and low IL-12 signaling [101]. The evidence for an immunoinhibitory Treg–pDC interaction in HCC patients has been substantiated by a working group from Rotterdam: CD4+ FoxP3- IL-13- IL-10+ Treg1 cells (CD49b+ and LAG-3+) inhibited T cell responses in an IL-10 dependent manner. This mechanism was stimulated by pDCs that were exposed to tumor-derived factors via the inducible costimulatory ligand (ICOS-L) [102]. As HCC patients have an increased prevalence of tumor-infiltrating ICOS+ Tregs and this subset is regarded as a potent immunosuppressor in HCC, these observations warrant further investigation of ICOS signaling inhibition [103].

(iii) It has been observed that DCs, and monocytes and B cells from HCC tumors, express the respective ligands for the inhibitory receptors programmed cell death protein 1 (PD-1), T cell immunoglobulin and mucin-domain containing-3 (TIM3), lymphocyte-activation gene 3 (LAG3) and CTLA-4, for which CD4+ and CD8+ T cells were significantly enriched in HCC patients. An antibody-mediated inhibition of this inhibitory signaling resulted in a restoration of CD8+ and CD4+ tumor-infiltrating lymphocyte proliferation and cytokine production [104].

### 4.2. Tumor Microenvironment

The tumor microenvironment (TME) exerts a significant influence on the tolerogenic immune response towards HCC. It encompasses myeloid-derived suppressor cells (MDSCs), tumor-associated macrophages (TAMs), cancer-associated fibroblasts (CAFs) and Tregs. The term MDSCs includes monocytes and relatively immature neutrophils, with the former differentiating into TAMs and infDCs in the TME [105]. They are potent inhibitors of the T cell response, and their proangiogenic properties directly encourage tumor growth [105,106]. Importantly, MDSCs accumulate in the liver during both hepatic and extrahepatic diseases and can be induced from myeloid cells by HSC [107]. Their presence is associated with a higher tumor burden, a higher rate of metastases, angiogenesis and an antiapoptotic effect on tumor cells. Additionally, their recruitment of CCL2-expressing tumor-associated neutrophils (TANs) promotes progression of HCC [52]. In vitro experiments have demonstrated an impaired DC maturation, antigen uptake, migration and induction of T cell IFNγ production in the presence of high numbers of MDSCs [108]. Furthermore, MDSCs directly inhibit cytotoxic T cells by producing arginase, reactive oxygen species, inducible nitric oxide synthase and IL-10, but also by competing with DCs and other APCs for cysteine [109,110]. Furthermore, MDSCs induce the differentiation of CD4+ CD25+ Foxp3+ Tregs in HCC patients [111].

### 4.3. Tregs

Low intratumoral Treg numbers in combination with a high number of activated CD8+ cytotoxic cells have been associated with a favorable OS and disease-free survival (DFS) in HCC patients [112]. In HCCs arising on the background of cirrhosis, the number of tumor-infiltrating Tregs was elevated in comparison to other etiologies [113]. Circulating CD4+CD25+ Tregs are trafficked into the tumor by the liver-specific chemokine receptor CCR6 and attracted by CCL20, secreted by HCC cells [113]. In HCC lesions, a markedly increased number of Tregs with a terminally differentiated phenotype (CD69+HLA-DR^high^) has been observed in comparison to nontumorous tissue, especially in poorly differentiated tumors and tumors with vascular invasion, and identified as an independent prognostic factor of OS and DFS [112,113,114]. In vitro, their depletion improved the proliferation of HCC tumor-associated antigen (TAA)-specific CD8+ T cells, but did not restore CD8+ functionality, as deduced from their persistently low IFN-γ production [114]. The main suppressive mechanisms of Tregs encompass the secretion of inhibitory cytokines, cytolysis, metabolic disruption and suppression of DC function via CTLA-4 and IDO [115]. A further mechanism of suppression has recently been extrapolated from deep single-cell RNA sequencing data, which revealed that the signature gene layilin is upregulated on activated CD8+ T cells and Tregs from the TME and represses the CD8+ T cell functions in vitro [116].

Of note, a group of CD14+CTLA-4+ regulatory DCs (CD14+DCs) with a similar suppressive effect as Tregs on T cells has also been identified in HCC patients. These DCs inhibited the T cell response in vitro via CTLA-4-dependent IL-10 and IDO production and expressed high levels of PD-1 [117].

### 4.4. Lymphocyte Function in HCC

The cross-presentation of tumor antigens by DCs enables the priming of CD8+ cytotoxic lymphocytes and the subsequent identification and destruction of tumor cells [118,119]. While tumor antigens and danger mediators from the tumor microenvironment are readily available to hepatic DCs and other APCs, several mechanisms can hamper efficient antigen presentation or inhibit a subsequent T cell mediated immune response [95]. Defects of antigen cross-presentation and subsequent suppressed T cell responses are a central mechanism of cancer immunosurveillance failure [97]. As such, several working groups reported a compromised function of HCC-infiltrating CD4+ Th1 cells and an enrichment of exhausted CD8+ T cells and Tregs in the TME [114,116,120]. Consequently, a predominance of a Th2-like cytokine profile and a concomitant decrease of Th1 cytokines were associated with negative prognostic parameters like progression to venous metastases [121].

CD8+ T cell responses against specific TAAs are considered to be a potential immunological driving force against HCC. These responses, however, can only be detected at very low frequencies in HCC patients, with a similar number of TAA-specific CD8+ T cells in healthy controls and in HCC patients [122]. In early-stage HCC and in patients with favorable outcomes, a wider breadth of CD8+ T cell responses targeting TAAs was noted. Still, these cells had a defective cytotoxic function with a lower IFN-γ production [114]. Recently, these functional limitations have been linked to a naïve CD8+ cell phenotype and the absence of exhausted TAA-specific CD8+ T cells, suggesting inefficient induction and restricted antigen recognition [122]. A plethora of influences hampers the cytotoxic antitumor response, such as the suppression of CD8+ T cells through Tregs, tumor-induced hypoxia, lactic acid accumulation, the secretion and expression of immunomodulatory mediators (IL-10, Fas/FasL, CXCL17, VEGF and IDO) and the weakened stimulation by APCs and CD4+ T cells [123,124].

The research efforts of the last decades regarding the role of the APC–T cell interaction have led to the understanding that inhibitory receptors on T cells, so-called immune checkpoints, for example PD-1, CTLA-4, TIM3 and LAG3, hamper the T cell response upon ligand binding. Ligands for immune checkpoints are not only expressed by tumor cells, but also by myeloid cells like DCs, TAMs, macrophages and neutrophils. The latter have been shown to upregulate programmed cell death 1 ligand 1 (PD-L1) expression in response to IL6-STAT3 pathway signals from the TME, namely from CAFs [125].

A hyporesponsive state of T cells typically arises in a chronic inflammatory environment and is termed “exhaustion” [126]. Exhausted TAA-specific CD8+ T cells with an elevated PD-1, TIM3 and LAG3 expression, upregulated activation markers and lowered levels of granzyme B and effector cytokines have been described in HCC patients [104]. Consistently, a low expression of different checkpoint molecules, reflective of intact cytotoxic and Th1 function and a low number of dysfunctional exhausted T cells, has been associated with an improved OS and recurrence-free survival in HCC [127,128].

The vascular abnormalities inherent to fibrosis seem to further impair hepatic immune surveillance. In 2015, the working group of Iannacone and Guidotti published the results of a murine advanced imaging study revealing that CD8+ effector T cells recognize hepatic antigens by extending cellular protrusions through endothelial fenestrae, a mechanism that is blocked by sinusoidal defenestration in fibrotic remodeling. Thus, antigen experienced CD8+ effector T cells may be incapable of recognizing transformed hepatocytes in the setting of fibrosis despite previous antigen presentation and activation [65].

## 5. Immunological Therapeutic Approaches

**Figure 1:**
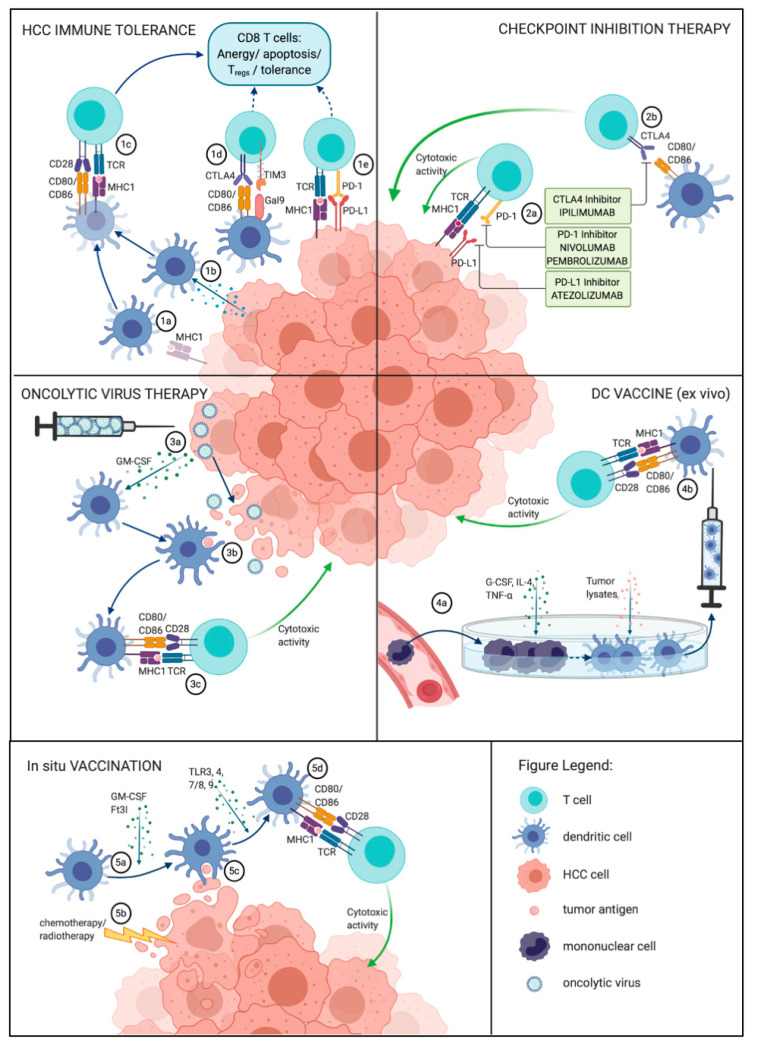
Dendritic cell (DC)–T cell-tumor interactions as targets of hepatocellular carcinoma (HCC) immunotherapy. HCC tumors employ various strategies of immune evasion. One of them is induction of immature or dysfunctional DCs whose antigen presenting capacity results in a tolerogenic direction of the immune response. (1a) Downregulation of tumor antigens results in (1b) antigen presentation by immature DCs with an instable DC–T cell interaction, leading to a failure to cross-prime CD8 cells and subsequent T cell anergy and tumor tolerance. Similarly, (1c) tumors can directly induce DC immaturity by secreting mediators like IL-10, VEGF and TGF-β. (1d) DCs can actively induce a tolerogenic response by expressing ligands of checkpoint pathways like CTLA-4 or TIM3 while downregulating stimulatory signals, inducing T cell anergy or Tregs, which in turn hamper cytotoxic T cell responses via IL-10. (1e) Tumor cells can evade CD8 control by expressing immune checkpoint ligands like PD-L1 and directly blocking cytotoxic T cells. Checkpoint inhibition of (2a) tumor cells or (2b) antigen-presenting cells leads to the inhibition of the inhibitory signals and results in an unleashed cytotoxic activity. Oncolytic viruses (3a) are injected peripherally and selectively infect HCC cells. Subsequently, a phase of replication with GM-CSF production release follows, GM-CSF recruits APCs to the tumor. (3b) Virus-induced immunogenic cell death results in a release of tumor antigens, which are phagocytized by the recruited DCs, triggering (3c) T cell priming and a systematic cytotoxic antitumor response. For an ex vivo vaccine (4a) mononuclear cells are isolated from the patient‘s blood and incubated with activating signals (granulocyte-colony stimulating factor (G-CSF), IL-4 and TNF-*α*) and tumor antigens, then transferred back into the patient. (4b) The injected DCs prime CD8+ lymphocytes and trigger an antitumor response. In situ vaccination: (5a) DCs are recruited with, e.g., Ft3l, while (5b) tumor mass is destroyed with radio- or chemotherapy, so that tumor antigens are readily available for (5c) phagocytosis. (5d) Maturation signals such as TLR3 induce antigen presentation to prime CD8+ T cells. A cytotoxic activity ensues. Created with BioRender.com.

Aiming to restore an efficient antitumor response, several promising immunological techniques targeting the DC–CD8+ T cell axis have been developed, ranging from adoptive immunotherapy and immune checkpoint inhibition to DC-based vaccines (Figure 1) [129].

### 5.1. Immune Checkpoint Inhibition

Immune checkpoint inhibitors were celebrated as the “breakthrough of the year” in 2013 by Science magazine and in 2018, JP Allison and T. Honjo were jointly awarded the Nobel Prize in Physiology or Medicine for their discovery of negative immune regulation inhibition for cancer therapy [130,131]. Antibodies against immune checkpoints (such as PD-1, CTLA-4) disrupt coinhibitory T cell signaling, thus reactivating the immune response against tumor cells [132]. Checkpoint inhibitors have dramatically improved the outcomes in several malignancies with a previously dismal prognosis, such as malignant melanoma, non-small cell lung cancer and mismatch repair-deficient colorectal carcinoma [133,134,135].

In HCC, response rates of 15–20% (compared to 2–3% on first-line Sorafenib [13,14]) to Nivolumab (anti-PD-1) were reported from a phase I/II trial (CheckMate 040) [15,16]. Based on data from this trial, the Food and Drug Administration (FDA) granted approval for Nivolumab as second-line therapy for HCC patients previously treated with Sorafenib [136]. On May 29, 2020, the FDA approved atezolizumab (anti-PD-L1 antibody) in combination with bevacizumab (anti-VEGF antibody) for patients with unresectable or metastatic HCC who have not received prior systemic therapy, based on the superior efficacy of this combination therapy compared to sorafenib in a phase 3 clinical trial [18]. While these results are encouraging in comparison to the data from previous HCC studies, it is clear that only a subset of patients benefits from immunotherapy, necessitating firstly, the identification of robust predictors of response to checkpoint inhibitor therapy in HCC and secondly, the evaluation of potential strategies to augment the response to immune checkpoint inhibition.

Several predictors of the response to the checkpoint blockade have been suggested, such as a high expression of PD-L1, high tumor baseline expression of immune-related genes, lymphocyte tumor infiltration, mismatch repair-deficiency and IFN-γ signaling [133,137,138]. In particular, compelling evidence that clinical responses to PD-1 inhibition occur in tumors with a pre-existing IFN-γ mediated cytotoxic response has been brought forward [139,140]. Thus, a “T cell inflamed” phenotype with lymphocyte infiltration and intense IFN-γ signaling has been termed “hot tumor”, while a lack of T cell infiltration is regarded as a “non-inflamed” or “cold tumor” [17].

Large-scale gene expression profiling revealed that about 27% of HCCs exhibit elevated markers of an inflammatory response, such as a high infiltration of immune cells, respective PD-1 and PD-L1 expression and enhanced cytolytic activity or IFN-γ signaling. This HCC group with an inflammatory response, in turn, was divided into subgroups based on the TME with about 65% belonging to an active immune response subtype with an activated adaptive T cell response and about 35% exhibiting an exhausted immune response, characterized by the presence of immunosuppressive signals like TGF-β and M2-type macrophages. The first group had a significantly better OS. Particularly the active immune response group thus resembled the immunological landscape of melanomas amenable to checkpoint inhibition therapy [140]. However, the majority of HCCs are not enriched for inflammatory TME responses, thus constituting a group of tumors with potentially little response to checkpoint inhibition.

While a subset of patients—for which predictive biomarkers in HCC have to yet be clinically implemented and tested—shows an impressive response to checkpoint blockade, it is clear that a large group of HCC patients derives little benefit from checkpoint inhibition because of primary resistance [15]. Possible reasons include a poor immunogenicity of the tumor with defects of T cell priming by DCs. Thus, it has been demonstrated that BATF3-dependent DCs (cDC1) are essential for the response to therapy with anti-PD-1 and anti-CD137 [141]. Furthermore, TAMs have been shown to capture anti-PD-1 antibodies from CD8+ lymphocytes via the Fc domain, abrogating the checkpoint inhibition signal [142]. Additionally, the expression of alternative checkpoint markers and the upregulation of immunosuppressive markers (e.g., IDO) in the tumor environment can contribute to the resistance against checkpoint inhibitors [143,144]. Several strategies to improve the efficacy of checkpoint inhibitors have been suggested and investigated clinically, for example, the activation of the previously inert antigen presentation or the inhibition of several checkpoints at once (see below).

### 5.2. DC-Based Vaccines

Arguing that poor tumor immunogenicity and defective DC maturation result in an impaired priming of cytotoxic CD8+ T cells, the ex vivo exposure of DCs to tumor antigens with concomitant stimulatory signals has been proposed to trigger an antitumor response. Most studies on ex vivo DC vaccines employ a similar procedure: first, mononuclear cells are isolated from peripheral blood and transferred into cell culture. Then, DCs are stimulated with activating cytokines such as granulocyte-macrophage colony-stimulating factor (GM-CSF), IL-4 and TNF-α and the exposure to a specific antigen is induced, usually by incubation with either tumor lysates or TAAs, but methods of cell fusion or tumor-associated RNA or DNA transfection have also been described [129,145]. Subsequently, the activated DCs are transferred to the patient where the injection of mature, antigen-loaded DCs results in the induction of both CD8+ cytotoxic T cells and CD4+ Th cells, particularly IFN-γ-producing Th1 cells [129,146].

Alternative stimulation techniques of the DC–T cell axis are emerging, such as the intratumoral injection of allogeneic moDCs previously stimulated with proinflammatory factors [147]. Due to the highly individualized approach of ex vivo DC vaccines, an enormous effort and extensive resources are necessary for patient treatment. The concept of in situ vaccines circumvents this problem with the aim of recruiting and activating intratumoral, cross-priming DCs. Based on the principle of abscopal radiation effects, namely that the local destruction of tumor mass at one location can induce the immune-mediated reduction of systemic, non-irradiated tumor burden, local destruction of the tumor is combined with Fms-like tyrosine kinase 3 ligand (Flt3L; a DC growth factor) injection and an activation signal, e.g., a TLR3 agonist to induce mature, immunogenic DCs that present tumor antigens [148].

The realization that poorly immunogenic tumors do not respond sufficiently to checkpoint inhibition prompted the approach of inducing an endogenous antitumor response before the checkpoint blockade. As such, studies conducted in poorly immunogenic melanoma more than two decades ago showed that, while the tumors did not respond to anti-CTLA4, a combination with a GM-CSF-transduced cellular vaccine induced a tumor response [149]. Combinations of in situ vaccines with subsequent checkpoint inhibition has shown promising effects in other tumor entities [150], but its efficacy remains to be evaluated in HCC.

### 5.3. Oncolytic Immunotherapies

Oncolytic viruses selectively infect and lyse tumor cells, triggering an immunological response to TAA. GM-CSF production from infected tumor cells has been suggested as an additional immunostimulatory mechanism [151,152]. Initially encouraging reports in predominantly sorafenib-naïve HCC patients suggested a significantly improved OS for the application of the oncolytic pox virus vaccine JX-594. A polyclonal humoral immune response resulting in antibody-dependent cytotoxic cell death and Th cell engagement and the induction of tumor-specific T cells was reported [153]. However, a subsequent randomized multicenter Phase IIb trial failed to show a difference in OS for JX-594 as a second-line HCC therapy after sorafenib failure. In JX-594-treated patients, a T cell response against vaccinia, β-galactosidase and TAA was noted, however, without improving patient survival [151]. Hypothesizing that vaccinia virus-based immunotherapy-mediated immune activation may resolve the immunosuppressive TME and pave the way for a sorafenib response, the PHOCUS phase III trial (NCT02562755) investigated sorafenib versus vaccinia virus-based immunotherapy and subsequent sorafenib application [154]. However, the study was recently halted after the completion of an interim futility analysis [155]. Further studies employing other therapeutic agents, combinations of treatments and adjuvants and in different stages of disease are on the way (e.g., NCT03203005 and NCT03071094) [156].

A possible explanation for the discrepancy between initially encouraging results in mouse models and early human trials and the negative results in subsequent human studies may lie in the pitfalls of the translation from mice models to humans. As such, murine fibrosis/HCC models, which constitute the foundation of basic immunological science, usually cannot reflect the full spectrum of human HCC etiologies and pathophysiology, with CCL_4_ models considered most similar to the human disease [157]. Further, differences in T cell polarization, costimulatory signaling and some aspects of antigen presentation between mice and humans may also impair the direct translation of results from immunological research [158].

### 5.4. Combination of Checkpoint Inhibitors

The combination of different immune checkpoint inhibiting agents has been suggested early in the experience with checkpoint inhibition of melanoma, but long-term follow-up data are not yet available for most tumor entities [159]. The fourth cohort of the CheckMate 040 trial enrolled patients with advanced HCC previously treated with Sorafenib and administered a nivolumab-ipilimumab (anti-PD-1 and anti-CTLA-4, respectively) combination. This combination led to overall response rates of over 30% (compared to 14-15% for the Nivolumab monotherapy group of Sorafenib-experienced patients [16]) and for the most successful dosage combination of Nivolumab 1 mg/kg + Iplimumab 3 mg/kg, a median OS of 23 months [160]. Since then, several clinical trials of combined immune checkpoint therapy for early and advanced HCC have been initiated, for example as a first-line therapy (NCT03298451), in the neoadjuvant setting before resection (NCT03682276 and NCT03222076), or in combination with interventional therapy (NCT0363814 and, NCT02821754) [161].

In 2019, several studies have shown that the two most prominent checkpoint inhibitors, anti-CTLA4 and anti-PD-1/PD-L1 convey distinctly different effects: CTLA-4 inhibition relieves constraints on CD4+ T cell phenotypes and allows for the differentiation of inducible costimulator (ICOS)+ CD4 Th1 cells [162], while anti-PD-1/PD-L1 therapy predominantly influences CD8+ lymphocytes and induces a TCF7-mediated increase of memory precursor-like CD8+ T cells [163]. It has furthermore been demonstrated that an efficient checkpoint inhibitor-induced antitumor response requires the expression of MHC class II-restricted antigens by tumor cells, combined with a local activation of CD4+ T cells to recruit and activate CD8+ T cells [164,165]. Therefore, the underlying mechanism of the efficacy and synergistic effects of different checkpoint inhibitor combinations have been investigated. The combination of anti-CTLA4 and anti-PD-1/PD-L1 therapy reduced the fraction of exhausted (PD1hiTIM3hiLAG3hi) CD8+ T cells, which were found predominantly in the context of anti-PD-1 monotherapy [164,166]. Similarly, in HCC cell lines, combinations of immune checkpoint antibodies against PD-L1, TIM3 and LAG3, which were found to be upregulated on TAA-specific CD8+ and CD4+ T cells, had additive effects in restoring the T cell response [104]. Thus, new molecular evidence now supports the previously clinically observed benefits.

## 6. Conclusions and Future Perspectives

Identifying the role of DC–T cell interactions in liver cirrhosis and carcinogenesis may not only help to unravel the mechanisms of fibrogenesis, but also provide essential clues towards the therapy of chronic hepatic diseases and HCC. Hepatocellular carcinoma arises against the background of a highly deregulated immune milieu with constitutive inflammatory signaling, activation of usually quiescent cell types and an antitumor response that is impaired by T cell exhaustion and by the physiologically tolerogenic direction of hepatic immune responses.

After several decades of a futile search for an efficient systemic HCC therapy, the last decade has witnessed the clinical incorporation of new agents into the clinical practice at a breathtaking pace. DCs and T cells form an immunologic backbone of tumor defense that is amenable to therapeutic targeting by the checkpoint blockade and DC vaccines. The forward and reverse translation between clinical experience and basic research enabled the advancement of additive or complimentary approaches in this field. At the same time, careful consideration of observations from animal models is necessary to gauge the efficacy of these strategies on humans to avoid the aforementioned pitfalls of translation. An improvement of our understanding of the role of DCs and T cells in HCC holds promise to optimize patient selection for existing treatments through biomarker-embedded approaches, to develop new strategies to overcome tumor tolerance and to improve the overall outcomes of patients with HCC.

## Figures and Tables

**Table 1:** Overview of dendritic cell populations with a focus on T cell interactions.

Population		Marker		Properties/Function
		Human	Mice	
cDC	Type 1	CD141, CD8, BATF3, IRF8, Clec9a, XCR1, TLR3	CD103, CD8, BATF3, IRF8, Clec9a, XCR1, TLR3	CD8+ T cell activation and cross-presentation [30], TLR2, TLR4
	Type 2	CD1c, CD11b	CD11b, IRF4	T helper cell priming with polarization toward Th2 or Th17 and promotion of humoral immunity [30]. Overall rare, but most frequent DC type in the human liver [25]. Secrete IL-10 upon TLR4 stimulation and induce T cell hyporesponsiveness [25]. Further subsets can be distinguished based on CD5, CD163 and CD14 expression, one of them is a circulating inflammatory (CD5-CD163+CD14+) subtype [28]
Pdc (precursors)		CD303, CD304, CD4, CD123^high^, TLR-7, TLR9	CD11c+ B220+ Gr-1+, TLR7, CD45Rb^high^ [26,31]	Antiviral innate immunity: antiviral response with abundant type 1 IFN production, stimulation of B cells, NK cells and T cells, differentiate into mature dendritic cells with intense T cell interaction [26] and capable of cross-presentation [32].
moDC		DC-SIGN(+)		Monocytes adopt a dendritic function and morphology in the presence of lipopolysaccharides or Gram-negative bacteria [33]. Used in vitro to model the DC function.

## Abbreviations

ALD	Alcohol-related liver disease
APC	Antigen-presenting cell
BDL	Bile duct ligation
CAF	Cancer-associated fibroblasts
CCL	Chemokine (C-C motif) ligand
CCl_4_	Carbon tetrachloride
CCR	C-C chemokine receptor type
cDC	Conventional dendritic cell
CTLA	Cytotoxic T-lymphocyte antigen
DC	Dendritic cell
DFS	Disease-free survival
DTR	Diphtheria toxin receptor
ECM	Extracellular matrix
EGF	Epidermal growth factor
FDA	Food and Drug Administration
Flt3L	Fms-like tyrosine kinase 3 ligand
GM-CSF	Granulocyte-macrophage colony-stimulating factor
GRO-α	Growth-related oncogene α
HCC	Hepatocellular carcinoma
HLA	Human leukocyte antigen
HSC	Hepatic stellate cell
ICAM	Intercellular adhesion molecule
ICOS-L	Inducible co-stimulatory ligand
IDO	Indoleamine 2,3-dioxygenase
IL	Interleukin
infDC	Inflammatory dendritic cell
LAG3	Lymphocyte-activation gene 3
LPS	Lipopolysaccharides
LSEC	Liver sinusoidal cell
MCD	Methionine/choline-deficient
MDSC	Myeloid-derived suppressor cells
MHC	Major histocompatibility complex
MMP	Matrix metalloproteinase
moDC	Monocyte-derived dendritic cell
NASH	Nonalcoholic steatohepatitis
NK	Natural killer
OS	Overall survival
PD-1	Programmed cell death protein 1
PD-L1	Programmed cell death 1 ligand 1
pDC	Plasmacytoid dendritic cell
PDGF	Platelet-derived growth factor
ROS	Reactive oxygen species
sFlt-1	Soluble vascular endothelial growth factor receptor 1
TA	Tumor-associated antigen
TAP	Transporter associated with antigen processing
TGF	Transforming growth factor
TAM	Tumor-associated macrophage
TIM1	T cell immunoglobulin and mucin-domain containing-3
TIMP1	Tissue inhibitor of metalloproteinases
TLR	Toll-like receptor
TME	Tumor microenvironment
TNF	Tumor necrosis factor
Tregs	Regulatory T cells
VAP1	Vascular adhesion protein 1
VCAM	Vascular cellular adhesion molecule
VEGF	Vascular endothelial growth factor
